# Impact of microglia isolation and culture methodology on transcriptional profile and function

**DOI:** 10.1186/s12974-024-03076-w

**Published:** 2024-04-08

**Authors:** Mark Mizrachi, Betty Diamond

**Affiliations:** 1 Feinstein Institutes of Molecular Medicine, Feinstein Institutes for Medical Research, 350 Community Drive, Manhasset, NY 11030 USA; 2 Donald and Barbara Zucker School of Medicine at Hofstra/Northwell, 500 Hofstra Blvd, Hempstead, NY 11549 USA

## Abstract

**Background:**

Microglial isolation and culturing methods continue to be explored to maximize cellular yield, purity, responsiveness to stimulation and similarity to in vivo microglia. This study aims to evaluate five different microglia isolation methods—three variants of microglia isolation from neonatal mice and two variants of microglia isolation from adult mice—on transcriptional profile and response to HMGB1.

**Methods:**

Microglia from neonatal mice, age 0–3 days (P0–P3) were isolated from mixed glial cultures (MGC). We included three variations of this protocol that differed by use of GM-CSF in culture (No GM-CSF or 500 pg/mL GM-CSF), and days of culture in MGC before microglial separation (10 or 21). Protocols for studying microglia from adult mice age 6–8 weeks included isolation by adherence properties followed by 7 days of culture with 100 ng/mL GM-CSF and 100 ng/mL M-CSF (Vijaya et al. in Front Cell Neurosci 17:1082180, 2023), or acute isolation using CD11b beads (Bordt et al. in STAR Protoc 1:100035, 2020. https://doi.org/10.1016/j.xpro.2020.100035). Purity, yield, and RNA quality of the isolated microglia were assessed by flow cytometry, hemocytometer counting, and Bioanalyzer, respectively. Microglial responsiveness to an inflammatory stimulus, HMGB1, was evaluated by measuring TNFα, IL1β, and IFNβ concentration in supernatant by ELISA and assessing gene expression patterns using bulk mRNA sequencing.

**Results:**

All five methods demonstrated greater than 90% purity. Microglia from all cultures increased transcription and secretion of TNFα, IL1β, and IFNβ in response to HMGB1. RNA sequencing showed a larger number of differentially expressed genes in response to HMGB1 treatment in microglia cultured from neonates than from adult mice, with sparse changes among the three MGC culturing conditions. Additionally, cultured microglia derived from adult and microglia derived from MGCs from neonates display transcriptional signatures corresponding to an earlier developmental stage.

**Conclusion:**

These findings suggest that while all methods provided high purity, the choice of protocol may significantly influence yield, RNA quality, baseline transcriptional profile and response to stimulation. This comparative study provides valuable insights to inform the choice of microglial isolation and culture method.

**Supplementary Information:**

The online version contains supplementary material available at 10.1186/s12974-024-03076-w.

## Introduction

**Fig. 1:**
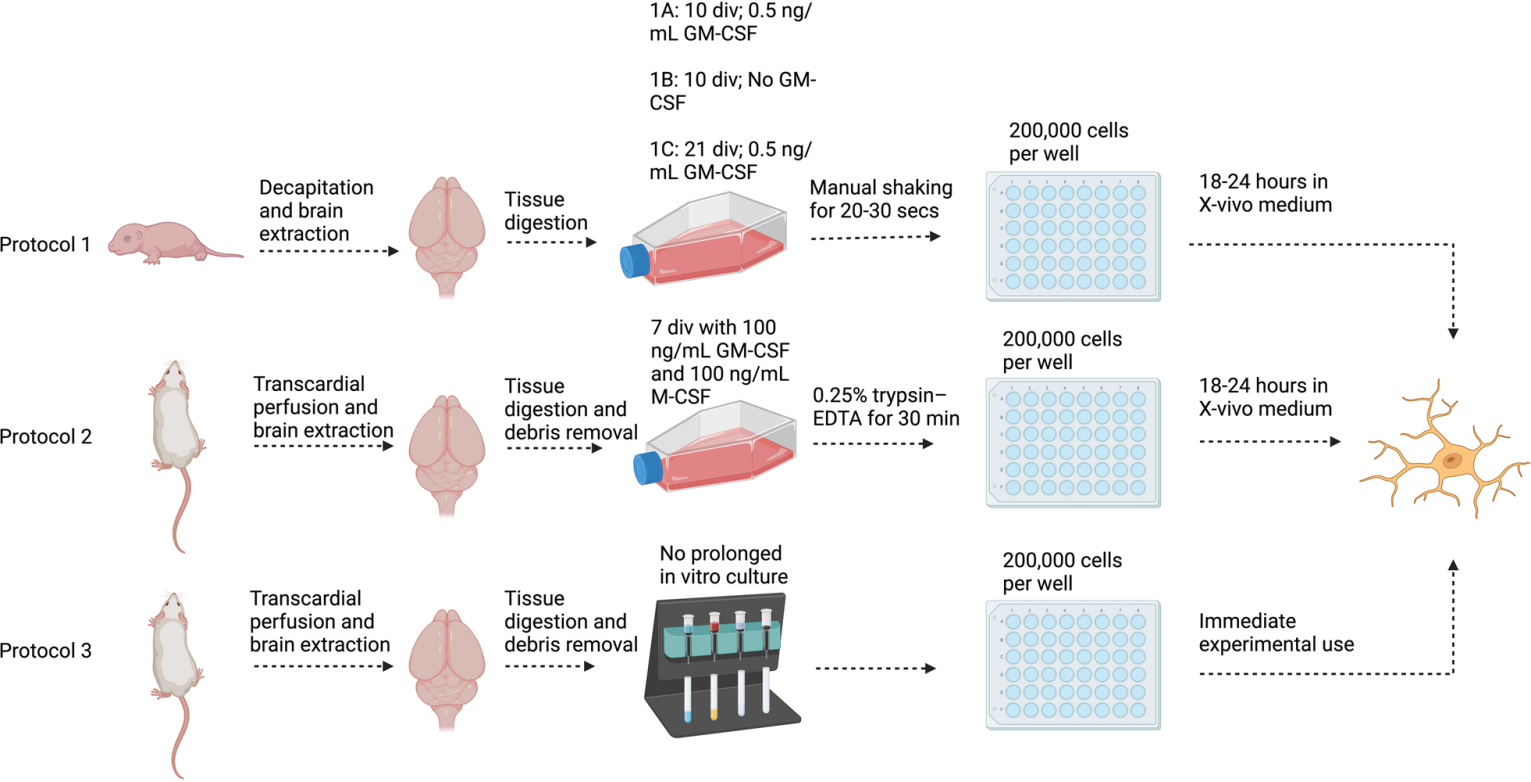
Overview of microglial isolation protocols. **A** Protocol 1 involves manually shaking microglia from MGCs sourced from p0-p3 pups with 3 variations: (1A) 10-day culture with 0.5 ng/mL GM-CSF, (1B) 10-day culture without GM-CSF, and (1C) 21-day culture with 0.5 ng/mL GM-CSF. **B** Protocol 2 involves microglia isolated by adhesive properties from a whole brain suspension, by replacing media after 3 h, then cultured for 7 days with 100 ng/mL GM-CSF and M-CSF. **C** Protocol 3 involves adult microglia isolated using anti-CD11b magnetic beads, and then immediately used for experimentation. Created with Biorender.com

Microglia, the resident immune cells of the central nervous system (CNS), are instrumental in maintaining homeostasis and responding to pathogens, as well as neuronal stress or injury [30]. They have a variety of functions, including phagocytosis, synaptic pruning, and secretion of cytokines. However, our understanding of microglial biology and its complex interplay with CNS pathologies remains limited, highlighting the necessity for reliable in vitro models to study their behavior in a controlled environment. This remains a challenge due to variations in isolation and culture techniques which may substantially influence the yield, purity, viability, and activation state of microglia. In this study, we asked whether there were critical differences in these parameters using three variations of isolating and culturing microglia from mixed glial cultures (MGC) from neonatal mice (P0–P3) and two methods for obtaining microglia from adult mice (as shown in Fig. 1). The MGC protocols from neonates differ with respect to the presence or absence of granulocyte–macrophage colony-stimulating factor (GM-CSF) and the duration of the culture period (Fig. 1A). The adult culture protocols differ in method of isolation and time in culture prior to stimulation [4, 35] (Fig. 1B, C).

Over the years, a number of papers have described protocols for microglia preparation from MGC [4, 8, 9, 17, 19, 22, 24, 34]. To enhance microglia yield, Hu et al. and Krabbe et al. opted to use conditioned medium from fibroblasts. Esen and Kielian et al. demonstrated that the inclusion of 0.5 ng/mL granulocyte–macrophage colony-stimulating factor (GM-CSF) in MGC improves microglial yield while preserving microglial identity and responsiveness. Others reported that higher concentrations of GM-CSF, more than 5 ng/mL, alter responsiveness to stimuli [21, 32]. We opted to compare microglia from neonatal MGCs using either no GM-CSF or 0.5 ng/mL GM-CSF.

The duration of in vitro culture is another factor to consider when studying microglial. The phenomenon of ‘culture shock,’ whereby cells change their behavior due to the stress of extended time in a non-physiologic environment, may compromise the translatability of phenotypic and functional studies to the in vivo environment [6]. However, maintaining MGCs for a longer period allows for a greater yield of microglia from a smaller number of animals. It has been previously published that microglia can be isolated from MGC after up to 28 days in culture [13]. We compared microglia separated from MGCs after 10 days culture to microglia separated from MGCs after 21 days of culture, to investigate the impact of the length of culture time on microglial response.

We adapted a previously described protocol [31, 35, 38], which involves a 7-day culture of adult microglia with 100 ng/mL GM-CSF and 100 ng/mL macrophage colony-stimulating factor (M-CSF). During this 7-day culture, microglia develop a ramified, adherent, reactive morphology in vitro [35]. We included an additional step, whereby non-adherent contaminating cells were removed using a lower concentration trypsin and discarded to enhance purity, prior to microglial separation with higher concentration trypsin, as described in “Methods”.

We also included a protocol for acute isolation using CD11b beads, as previously described [4, 15, 16, 36]. Immediate isolation and treatment negates the effects of culture shock. However, the yield and viability of microglia obtained with this protocol is significantly less than what is obtained using the in vitro culture protocols from neonatal mice [35].

High Mobility Group Box 1 (HMGB1) is a potent pro-inflammatory mediator which is implicated in various pathological conditions of the CNS [1, 11, 28, 29]. It resides in the nucleus of all cell types as a chromatin-associated protein, but can be actively secreted as a damage-associated molecular pattern (DAMP) in the setting of cellular stress and injury [33]. Our prior work and studies from others have shown that neurons activated through the *N*-methyl-d-aspartate receptor (NMDAR) secrete HMGB1 [10, 37], which activates microglia, triggering an inflammatory response that contributes to neuronal damage in the setting of neuropsychiatric disease [7, 18]. Consequently, the response of microglia to HMGB1 stimulation was assessed to provide insight into potential differences in function of microglia obtained with the different protocols.

## Methods

### Protocol 1: isolation and culturing of primary microglia from neonatal mice

Primary microglia were isolated from postnatal P0–3 C57BL/6 mouse neonates. Neonates were euthanized by decapitation. Brains were then excised under aseptic conditions and dissociation was performed using the Neural Tissue Dissociation kit according manufacturer’s instructions (Miltenyi Biotec, Bergisch Gladbach, Germany). The resulting pellet was gently resuspended in complete DMEM (containing 4.5 g/L glucose, 10% FBS, 1% penicillin–streptomycin) and was filtered through a 70 μm cell strainer (Corning, NY, USA) to eliminate any clumped cells or tissue debris. Based on the intended protocol variation, cell suspensions were treated as follows:

- Protocol 1A: Cells were cultured in complete DMEM supplemented with 0.5 ng/ml recombinant mouse GM-CSF (R&D Biosystems, Minneapolis, USA) for 10 days.
- Protocol 1B: Cells were cultured in complete DMEM (without GM-CSF) for 10 days.
- Protocol 1C: Cells were cultured in complete DMEM supplemented with 0.5 ng/ml recombinant mouse GM-CSF for 21 days.

For each of the above protocols, cell suspensions were derived from 5 mice. Cells were plated onto a 175 cm^2^ flask, and medium was changed every 3 days. Upon reaching confluence, on day 10, microglia were dislodged and harvested from culture by manual shaking for 20–30 s. The medium containing the detached microglia was collected, and cell count was determined. This was followed by centrifugation at 400*g* for 10 min to pellet the cells. The microglial pellet was resuspended in serum-free X-VIVO medium (Lonza Biosciences, Walkersville, MD), and plated into 48-well Falcon tissue culture plates (Fisher Scientific, Waltham, Massachusetts, U.S.) at 200,000 cells per well. X-VIVO medium is a serum-free medium, which lacks exogenous growth factors, artificial stimulators of cellular proliferation, or undefined supplements. It has been used for myeloid cell growth as well as microglia cultivation [7, 12, 25]. Cells were allowed to adhere overnight and were ready for experimentation the subsequent day. At least 3 biological replicates for each condition were performed, with each replicate representing microglia isolations derived from distinct litters of mice.

### Protocol 2: isolation and culturing of primary microglia from adult mice by adherence properties

C57BL/6 mice, at 6–8 weeks, were administered a lethal dose of Euthasol followed by transcardial perfusion with 0.9% ice cold heparinized saline. Brains were excised under aseptic conditions, and tissue dissociation was performed using the Neural Tissue Dissociation Kit, according to the manufacturer’s instructions. Following dissociation, myelin debris was separated and removed using Debris Removal Solution (Miltenyi Biotec, Bergisch Gladbach, Germany), according to manufacturer’s instructions. The cell pellet was resuspended in pre-warmed complete DMEM and cells from 3 brains were pooled together and plated on a T75 flask. After a 3-h incubation period to allow adherence, the medium was gently replaced to discard non-adherent cells. The medium was supplemented with 100 ng/mL macrophage colony-stimulating factor (M-CSF) and 100 ng/mL GM-CSF on the following day. Medium was changed on day 4 with complete DMEM supplemented with M-CSF and GM-CSF, and on day 7, contaminating cells were collected using 0.05% trypsin- ethylenediaminetetraacetic acid (EDTA) for 10 min and discarded. Contaminating cells adhered less strongly to the flasks than microglia when exposed to 0.05% trypsin, so this step allowed for enhanced purity. Microglia were then obtained by incubating flasks in 0.25% trypsin–EDTA for 30 min. Trypsin was quenched with complete DMEM, and cells were removed from the flasks and centrifuged at 400*g* for 10 min, followed by resuspension in X-VIVO serum-free medium (Lonza Biosciences, Walkersville, MD). Cells were plated in 48-well dishes (Falcon) at 200,000 cells per well, and they were ready for experimentation the following day.

### Protocol 3: acute isolation of microglia from adult mice using anti-CD11b MicroBeads

C57BL/6 mice, at 6–8 weeks, were administered a lethal dose of Euthasol followed by transcardial perfusion with 0.9% ice cold heparinized saline. Brains were excised under aseptic conditions, and tissue dissociation was performed using the Neural Tissue Dissociation Kit, according to the manufacturer’s instructions. Following dissociation, myelin debris was separated and removed using Debris Removal Solution, according to the manufacturer’s instructions. CD11b-positive cells were enriched using anti-CD11b MicroBeads (Miltenyi Biotec, Bergisch Gladbach, Germany), according to manufacturer’s instructions. The resulting cell:bead suspension was centrifuged at 400*g* for 10 min and resuspended in X-VIVO medium (Lonza Biosciences, Walkersville, MD), or X-VIVO medium supplemented with 1 ug/mL HMGB1 for immediate stimulation.

### Cell culture treatment

HMGB1 was obtained as a generous gift from Kevin Tracey, MD, of the Feinstein Institutes for Medical Research. Cells were treated with HMGB1 for 4 h prior to harvesting for analysis of bulk mRNA sequencing and for 24 h prior to analysis of cytokines in cell culture supernatant by ELISA.

### RNA extraction

Total RNA was extracted from microglia using the Qiagen RNeasy RNA extraction kit (Qiagen) according to the manufacturer's instructions. Briefly, cells from each well were homogenized in RLT lysis buffer. The homogenate was then passed through QIAshredder spin columns to remove cellular debris, and RNA was purified using RNeasy spin columns. RNA was eluted in 30 μl RNase-free H_2_0.

### ELISA

Cell culture supernatant was collected and centrifuged at 500*g* for 5 min followed by separation of supernatant. The Duoset TNFα, Duoset IL-1β, and Duoset IFNβ ELISAs (R&D Biosystems, Minneapolis, MN) were performed on supernatant according to the manufacturer's instructions.

### Flow cytometry

Cells were washed in FACS buffer (1% BSA, 0.1% sodium azide in PBS), then incubated in FACS buffer containing functional viability dye (65-0866-14, ThermoFisher Scientific) along with anti-CD45 (1:80, BioLegend, clone 30-F11), anti-CD11b (1:200, BD Biosciences, clone M1–70) and anti-transmembrane protein 119 (Tmem119; 1:500, Abcam, clone 106-6) antibodies for 15 min at 4 °C in the dark. After staining, cells were washed with FACS buffer, and flow cytometry was performed using the BD LSRFortessa™ Cell Analyze. Data analysis was conducted using FlowJo.

### mRNA sequencing analysis

Gene read counts were obtained using featureCounts v1.5.0 [23], and normalized using the DESeq2 package (1.20.0) [26] with variance-stabilizing transformation (VST). Differential gene expression following HMGB1 treatment for each isolation protocol was assessed, using an adjusted p value < 0.05 and an absolute fold change of 1. Raw gene counts from the NCBI Gene Expression Omnibus GEO accession number GSE79819 [27] were downloaded and combined with our data, and we applied batch correction using combat-seq. For gene set enrichment analysis, a list of genes that were DEGs in both the Protocol 1 vs Protocol 3 comparison and the early microglia vs adult microglia comparison were compiled based on log2fold change > 2 and p value < 0.01 in both comparisons, giving us a list of 399 genes which was input into EnrichR [20]. The top 10 terms from GO Biological Processes 2023 were identified.

### Statistical methods

Statistical analyses were conducted using R for bulk mRNA sequencing data and GraphPad Prism for ELISA measurements. For neonate-derived cultures, each experimental group comprised three biological replicates from separate mouse litters. One-tailed t-tests were employed to compare cytokine secretion levels (TNFα, IL1β, IFNβ) between control and HMGB1-treated groups for each isolation method. Results are presented as median ± standard deviation.

### Data availability

All bulk sequencing data generated and analyzed during this study are publicly accessible in the Gene Expression Omnibus (GEO) repository. The datasets can be retrieved under the accession number GSE242683.

## Results

**Fig. 2:**
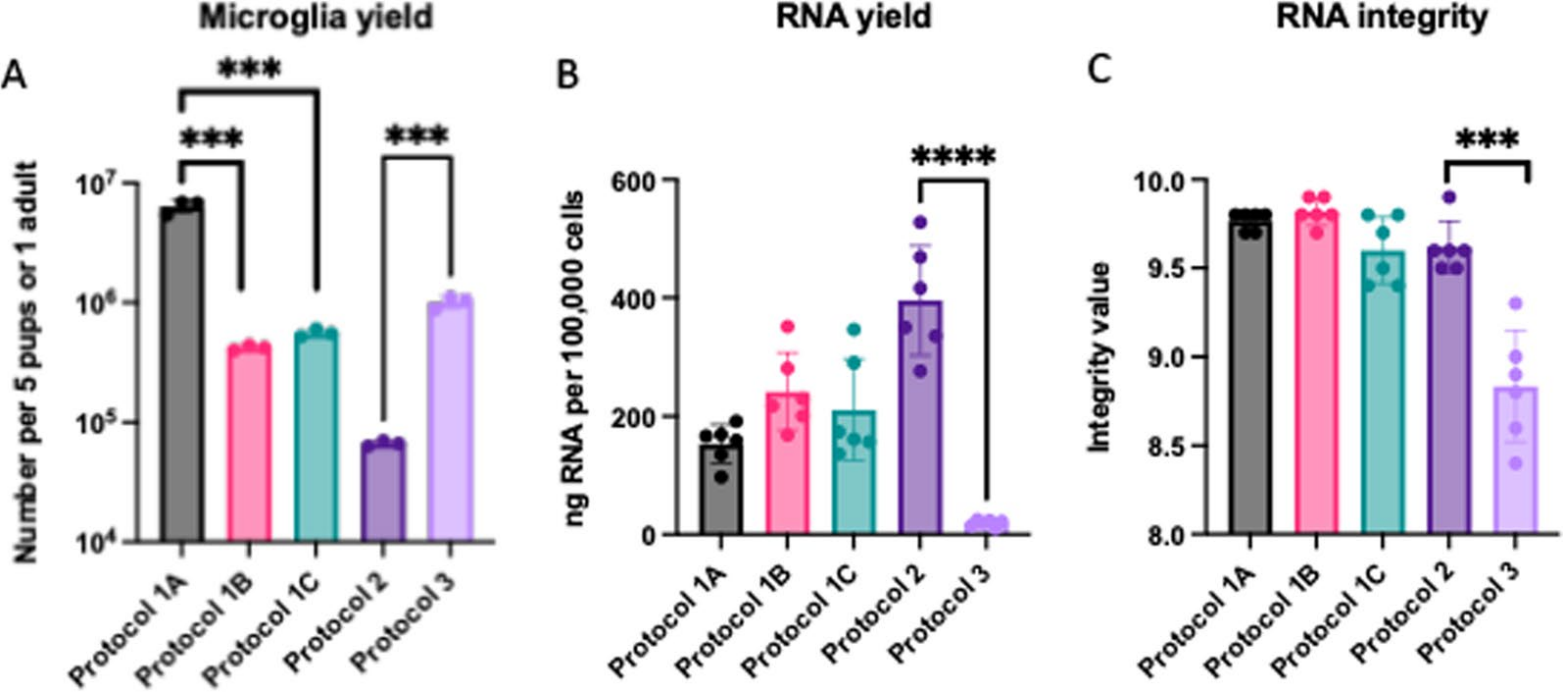
Analysis of microglial isolation metrics. **A**–**C** Bar graphs highlight microglial yield, RNA yield and RNA integrity across Protocols 1A, 1B, 1C, 2, and 3. **A** Total microglia yield as measured by hemocytometer, per 5 pups or 1 adult. **B** RNA yields from Nanodrop, displayed as ngRNA/100,000 lysed cells. **C** RNA integrity score assessed by Bioanalyzer. Asterisks indicate p values of most relevant comparisons, determined by a two-tailed t-test: *p < 0.05, **p < 0.01, ***p < 0.001, ****p < 0.0001

Microglial isolation efficacy was evaluated based on purity, yield, and RNA integrity (Fig. 2). Protocol 1A, 1B and 1C yielded microglia with similar purity, all exceeding 95%, Protocol 2 yielded 90.7% purity, and Protocol 3 yielded 91.7% purity, as confirmed by flow cytometry, with antibodies against CD11b and CD45. There was minimal contamination from macrophages (representative flow cytometry in Additional file 1: Figure S1). Protocol 1A led to the greatest yield, and between adult-derived cells, Protocol 3 surpassed Protocol 2 in yield (Fig. 2A). However, Protocol 2 resulted in superior RNA yield and integrity scores, compared to Protocol 3 (Fig. 2B, C). This reduction in RNA yield in Protocol 3 is likely due to the enzymatic digestion process, which may have exposed RNA to nucleases, leading to a lower RNA yield.

**Fig. 3:**
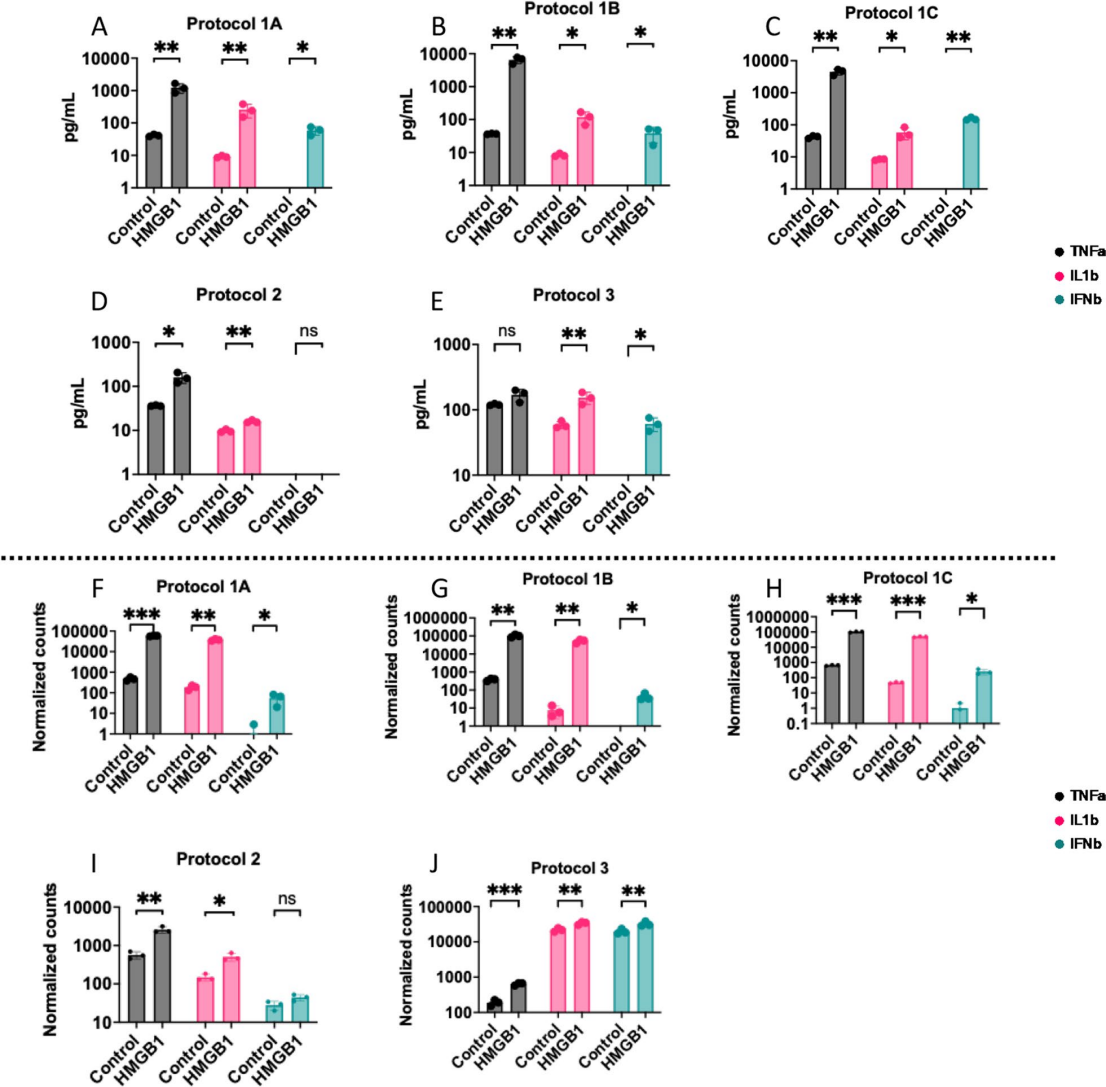
Differential cytokine secretion and transcription in response to HMGB1 stimulation in microglia derived from isolation protocols. **A**–**E** Cytokine secretion by microglia, quantified as concentration interpolated from a standard curve, 24 h post-HMGB1 stimulation. For each panel, grouped bar graphs represent concentration of TNFα, IL1β, and IFNβ in culture supernatant. **F**–**J** Transcription of the same cytokines as determined by bulk mRNA sequencing, displayed as normalized gene counts derived using DESeq2. Grouped bar graphs indicating transcription of TNFα, IL1β, and IFNβ. **A**, **F** Microglia derived from Protocol 1A. **B**, **G** Microglia derived from Protocol 1B. **C**, **H** Microglia derived from Protocol 1C. **D**, **I** Microglia derived from Protocol 2. **E**, **J** Microglia derived from Protocol 3. Dots represent microglia isolations derived from distinct litters of mice for neonatal mice or distinct adult mice

Microglial cytokine responses for 24 h HMGB1 treatment were consistent across neonate-derived cells (Fig. 3A–C). However, HMGB1 treatment resulted in TNFa secretion in Protocol 2 but not Protocol 3, whereas IFNβ was induced in Protocol 3, but not Protocol 2 (Fig. 3D, E). Similar patterns were observed for normalized gene expression from bulk sequencing data (Fig. 3F–J) after 4 h HMGB1 treatment; however, HMGB1 induced TNF mRNA expression in all protocols.

**Fig. 4:**
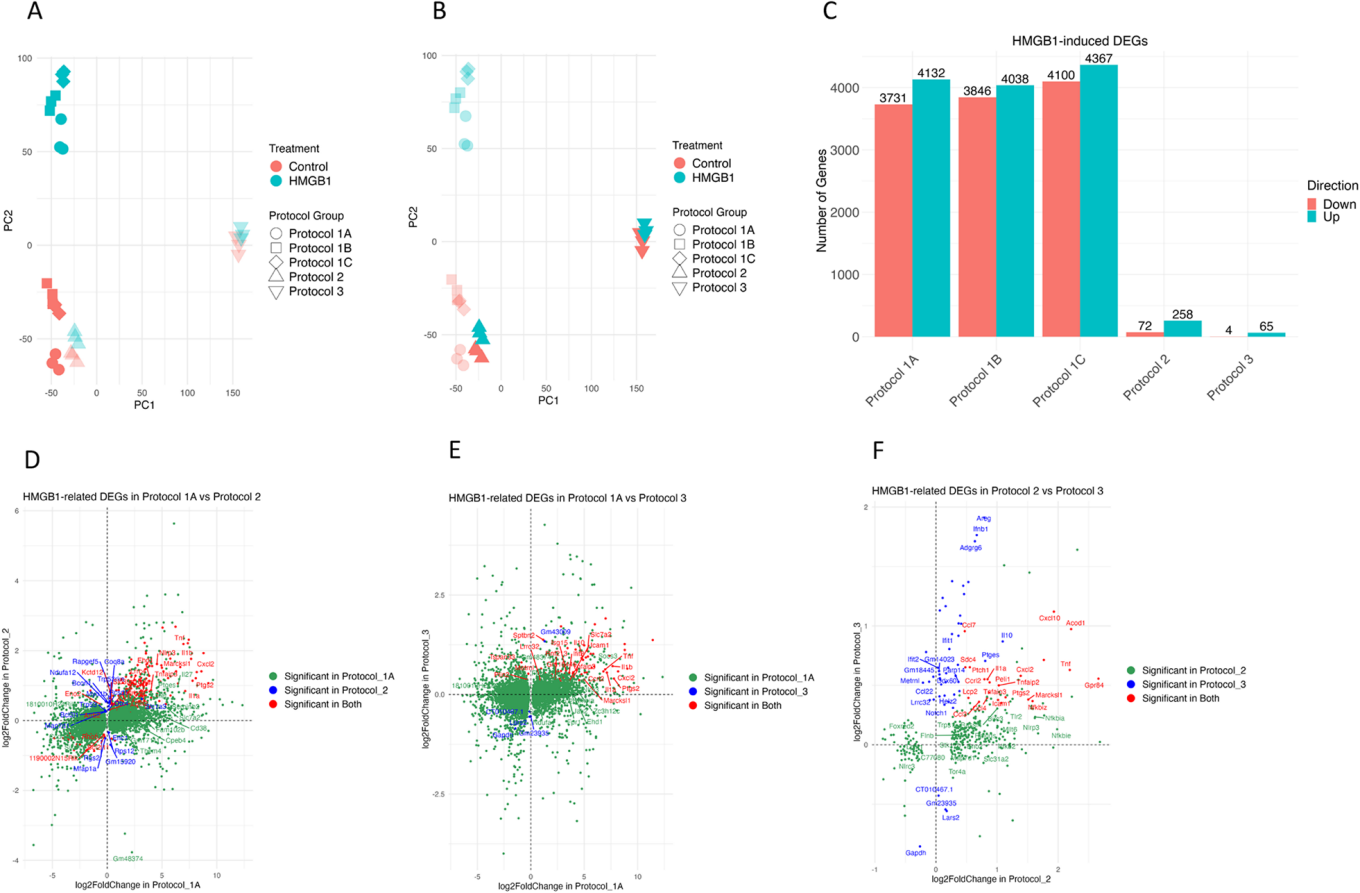
Effects of HMGB1 on transcriptomic profile of microglia from each protocol. **A**–**C** In a PCA scatter plot (**A**, **B**), microglial isolation protocols are represented by shapes: Circle (Protocol 1A), Square (1B), Diamond (1C), Triangle (2), and Upside-down triangle (3). Treatment colors are Red (Control) and Blue (HMGB1), with three replicates each. **A** Pup derived microglia are highlighted; **B** Adult derived microglia are highlighted. Grouped bar graphs (**C**) exhibit the number of DEGs after HMGB1 treatment across protocols, differentiated by Red (downregulated) and Green (upregulated) bars, with significance set at an adjusted p-value < 0.05. **D**–**F** Log2FoldChange plots display the top 250 genes, marking the 20 most significant. **D** compares Protocol 1A (X-axis) and 2 (Y-axis); **E** compares 1A (X-axis) and 3 (Y-axis); **F** compares 2 (X-axis) and 3 (Y-axis). Genes significant in the first protocol are Green, in the second are Blue, and in both are Red

Bulk mRNA sequencing revealed transcriptional signatures for each protocol. Protocol 3 was distinguished from the other protocols along PC1. PC2 reflected HMGB1 responsiveness, with greater effects in neonate-derived cells compared to adult-derived cells (Fig. 4A, B). Notably, microglia isolated using Protocol 2 more closely resembled Protocols 1A, 1B, and 1C than Protocol 3, in transcriptional profile (Fig. 4A, B), despite being derived from adult.

HMGB1 treatment resulted in up- and down-regulation of numerous genes in neonate-derived cells, greatly exceeding the responsiveness of adult-derived cells (Fig. 4C). Log2FoldChange plots elucidate gene-specific responses (Fig. 4D–F), for protocol 1A compared to protocol 2 (Fig. 4D), protocol 1A to protocol 3 (Fig. 4E), and protocol 2 to protocol 3 (Fig. 4F).

**Fig. 5:**
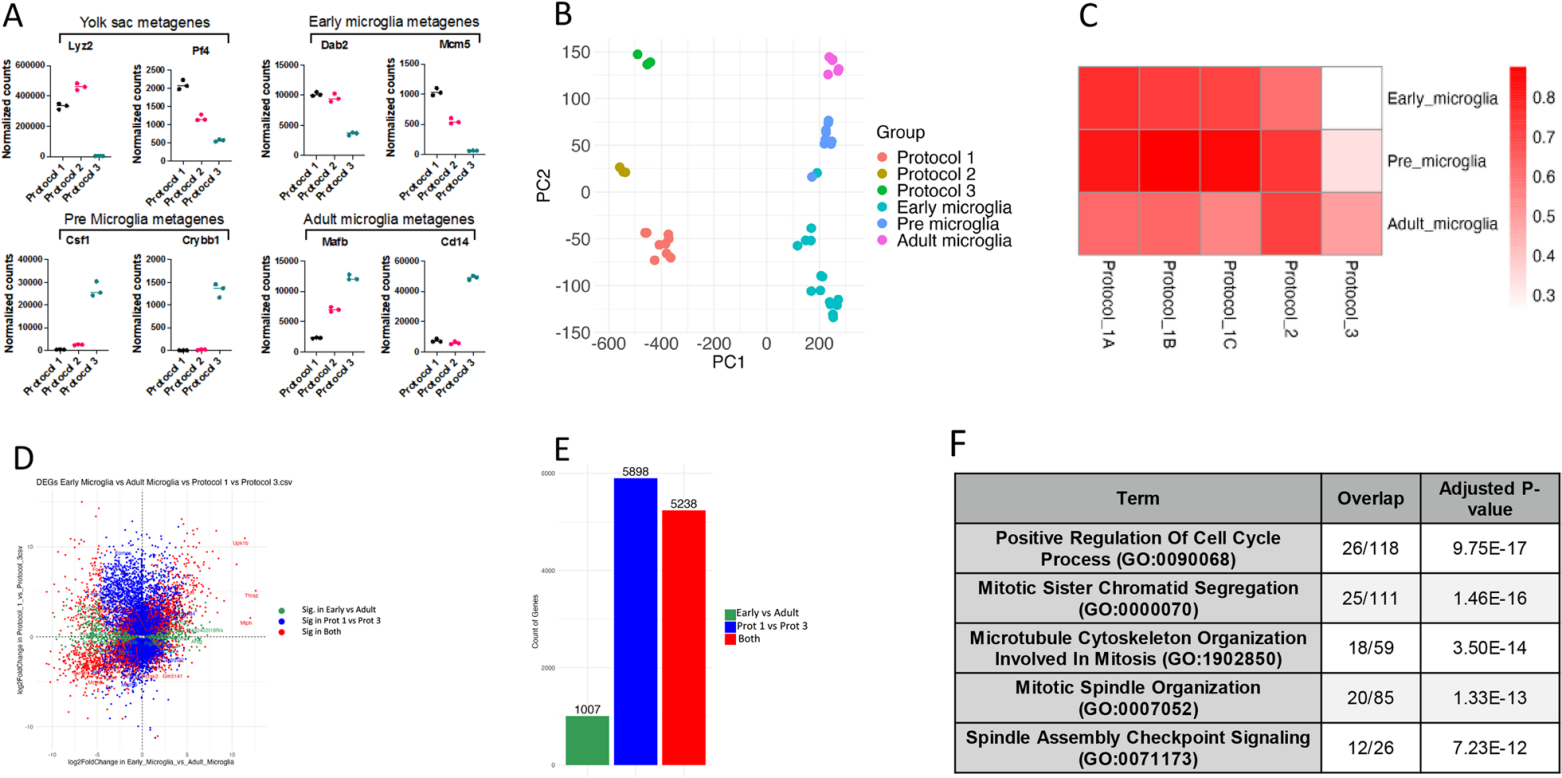
Transcriptomic profile of untreated microglia compared to developmental states. **A** Scatter dot plots showing the normalized gene counts for NMF metagenes identified in the external dataset, across our untreated conditions (Protocol 1, Protocol 2, Protocol 3), and corresponding to specific microglial developmental stages (Yolk sac, Early Microglia, Pre Microglia, Adult Microglia). **B** PCA plot illustrating the overlap between our bulk sequencing dataset for untreated conditions and external dataset. **C** Heatmap of Pearson correlation R^2^ values comparing five conditions, untreated, from our dataset (Protocol_1A, Protocol_1B, Protocol_1C, Protocol_2, Protocol_3) with those in the external dataset (Early_microglia, Pre_microglia, Adult_microglia); white corresponds to R^2^ = 0.3 and red to R^2^ = 0.8. **D** Log2 fold-change plot displaying DEGs comparing Protocol 1 vs Protocol 3 and Early Microglia vs Adult Microglia; green dots represent DEGs significant only in the early vs adult comparison (*p* < 0.05), blue dots are significant only in Prot 1 vs Prot 3 comparison(*p* < 0.05), and red dots are significant in both. **E** Bar graph depicting the number of significant DEGs unique to each comparison and those common to both; colors correspond to those in panel D. **F** Summary chart of Gene Ontology (GO) analysis conducted on genes with log2 fold-change > 2, and p < 0.01, in both our dataset comparing Protocol 1 with Protocol 3, and in the external dataset, comparing Early Microglia with Adult Microglia; analysis utilized EnrichR to identify GO Biological Process 2023 terms

Several metagenes have been shown to correspond to developmental stages—yolk sac, early microglia (day 14 or younger), pre microglia (E14 to P9) and adult microglia (4 weeks and older), using nonnegative matrix factorization (NMF) [27]. NMF is a method for identifying distinct molecular patterns that reduces the dimension of expression data from thousands of genes to a handful of metagenes [5]. We quantified the expression of these NMF-identified metagenes in microglia from Protocol 1, 2 and 3, and found that microglia from Protocols 1 and 2 had increased expression of metagenes associated with yolk sac and early microglia metagenes, whereas cells from Protocol 3 had increased expression of metagenes associated with pre microglia and adult (Fig. 5A, Additional file 1: Figure S2). We then integrated our dataset with the external dataset, sourced from “Microglia development follows a stepwise program to regulate brain homeostasis,” with GEO accession number GSE79819 [27] and analyzed the datasets together. Protocol 1 and 2 microglia align most closely with early microglia and pre-microglia from the external dataset, along PC2 (Fig. 5B), and Pearson correlation revealed that Protocol 1 and Protocol 2 had higher R^2^ values when compared to early and pre microglia than when compared to adult microglia (Fig. 5C). We then performed differential gene expression (DGE) analysis to identify which transcriptional patterns are responsible for protocol-dependent shifts in our dataset and were analogous to the developmental stage-dependent shifts in the external dataset. We compared DEGs between protocol 3 and protocol 1 and between adult and early microglia in the external dataset (Fig. 5D). 5238 of the DEGs between Protocol 1 and Protocol 3 were also identified as DEGs between early and adult microglia in the external dataset (Fig. 5E). We then identified all genes with a log_2_fold change greater than 2, and p-value less than 0.01, in protocol 1 compared to protocol 3, and early microglia compared to adult microglia. This generated a list of 399 genes, which we then submitted to EnrichR for gene set enrichment analysis. We identified the top ten GO Biological Process 2023 which corresponded to our gene list. This revealed a variety of cell cycle related terms as expected (Fig. 5F)

## Discussion

Microglia are the primary resident immune cells of the central nervous system, and they have a broad range of functions, including maintaining homeostasis, mediating neuroinflammation, and modulating neural circuitry [30]. Importantly, microglial gene expression in mice correlates closely with microglial gene expression in humans [14]. Therefore, a robust in vitro model is essential for studying human disease. In this study, we compared five protocols which can be used to isolate and culture microglia: three variations of isolation from MGC from neonates and two isolation protocols from adult. We analyzed each protocol for microglial yield, RNA yield/quality, baseline transcriptional profile, and response to HMGB1 stimulation. Our findings of greater than 90% purity across all isolation methods corroborate existing studies [4, 34, 35].

Our findings reveal that microglia from neonatal mice exhibit a greater number of differentially expressed genes in response to HMGB1 treatment than microglia from adult mice. This difference underscores the distinct behaviors of neonatal-derived microglia compared to adult microglia. Moreover, we demonstrated that all three variations of isolation protocols derived from MGCs were similar. However, the variation in gene expression profiles between Protocols 1, 2, and 3 prompts caution in generalizing results obtained from different protocols.

Prior research has demonstrated that microglia isolated from mice at different developmental stages exhibit unique transcriptional signatures, and metagenes have been identified which are associated with each developmental stage [5]. Although we did not include acutely cultured neonatal microglia in our experiments to compare directly with microglia from Protocol 3, we did find that expression of these metagenes differed across isolation protocols. Specifically, microglia from protocols 1 and 2 expressed metagenes associated with early and pre-microglial stages, whereas those from protocol 3 expressed metagenes associated with adult microglia. By integrating this external RNAseq dataset with our own, we further validated these observations. Collectively, our findings suggest that cultured microglia, regardless of originating from neonates or adults, adopt a transcriptional signature skewed towards earlier developmental stages. Future studies could investigate these effects more directly by comparing microglia derived from each protocol prior to and after culture.

A recent study by Cadiz et al. used scRNA seq to reveal that cultured microglia are heterogenous and deviate from in vivo microglia by undergoing a “culture shock” characterized by an activated transcriptional state [6]. Our analysis suggests that the described “culture shock” transcriptional state may resemble that of an earlier developmental stage.

It has previously been shown that mature microglia lose their signature gene expression rapidly after isolation, and this can be reversed by engrafting cells back into an intact brain [3]. Therefore, it is likely that the protocol-dependent genetic signatures identified here are reversible if given the right environmental stimuli. TGF-β, an important mediator of inflammation and embryogenesis, has been identified as a key molecule secreted by astrocytes and neurons that preserves the in vivo microglial transcriptional state [2].

Additionally, the use of acutely isolated microglia without culture has several limitations. The isolation process involves brain tissue dissociation, myelin removal, and microglia isolation using targeted antibodies conjugated to magnetic beads or fluorescent molecules, which can impact the microglial baseline transcriptional and functional state. In our studies, acutely isolated microglia yielded significantly lower RNA compared to other protocols (Fig. 2). Although culturing microglia has drawbacks, it allows recovery from isolation shock. Cold dissociation has been employed to reduce this shock, but it results in lower yields, reduced viability, and increased cell clumping, complicating its experimental use [4].

There is a need for careful consideration in the selection of microglial isolation and culturing methods, particularly when the focus of the study involves the role of microglia at different developmental stages or under different pathological conditions. Moreover, the differential expression of metagenes across isolation protocols and diverse responses to HMGB1 stimulation underscores the necessity for standardization in experimental designs, for reliable cross-study comparisons. Overall, acknowledging these nuances could enhance the rigor and interpretability of microglial studies.

In summary, this study evaluates five microglia isolation protocols, revealing high purity across all methods but with variations that have biological implications. We extend the concept of “culture shock” by demonstrating that observed transcriptional alterations parallel developmentally relevant states in microglia. Our findings serve as both a methodological guide and a contextual framework for understanding the biological nuances inherent in microglia isolation.

### Supplementary Information

**Additional file 1: Figure S1.** Flow cytometry of microglia-specific cell surface markers. A,B) Microglia isolated using protocol 1; C,D) Microglia isolated using Protocol 3; E,F) Cell suspension from whole brain. **Figure S2.** Scatter dot plots of normalized gene counts for NMF metagenes across all 5 conditions.

## Data Availability

All sequencing data generated and analyzed during this study are publicly accessible in the GEO repository. The datasets can be retrieved under the accession number GSE242683.
