# Supplementary material for: Impact of microglia isolation and culture methodology on transcriptional profile and function
Source: J Neuroinflammation. 2024 Apr 8;21:87. doi: 10.1186/s12974-024-03076-w (PMC11000335; doi:10.1186/s12974-024-03076-w)
Supplement: Supplementary file 1 — Additional file 1: Figure S1. Flow cytometry of microglia-specific cell surface markers. A,B) Microglia isolated using protocol 1; C,D) Microglia isolated using Protocol 3; E,F) Cell suspension from whole brain. Figure S2. Scatter dot plots of normalized gene counts for NMF metagenes across all 5 conditions. [file 12974_2024_3076_MOESM1_ESM.pptx]

## Slide 1
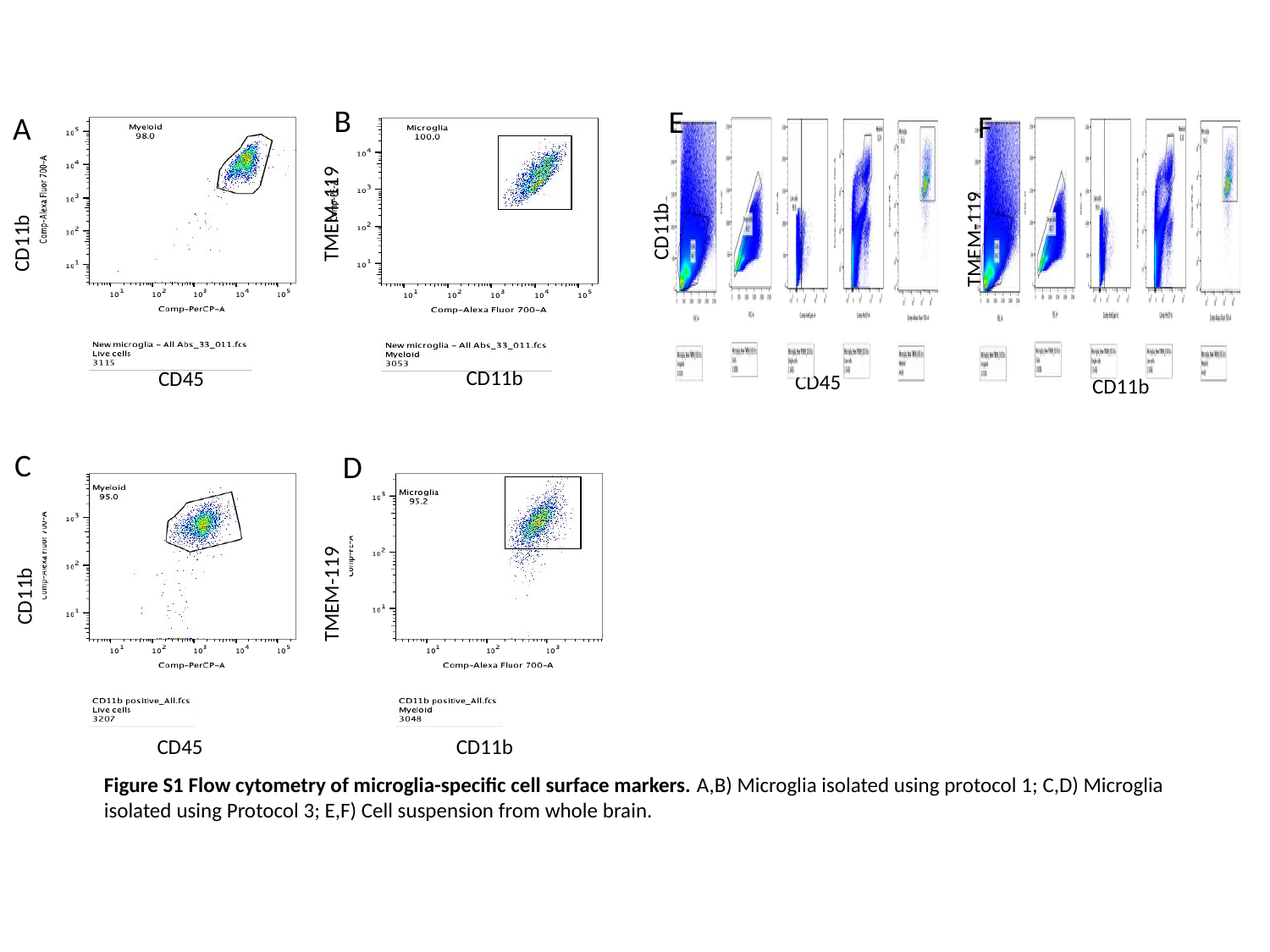

B
E
F
A
TMEM-119
CD11b
TMEM-119
CD11b
CD11b
CD45
CD45
CD11b
C
D
TMEM-119
CD11b
CD45
CD11b
Figure S1 Flow cytometry of microglia-specific cell surface markers. A,B) Microglia isolated using protocol 1; C,D) Microglia isolated using Protocol 3; E,F) Cell suspension from whole brain.

## Slide 2
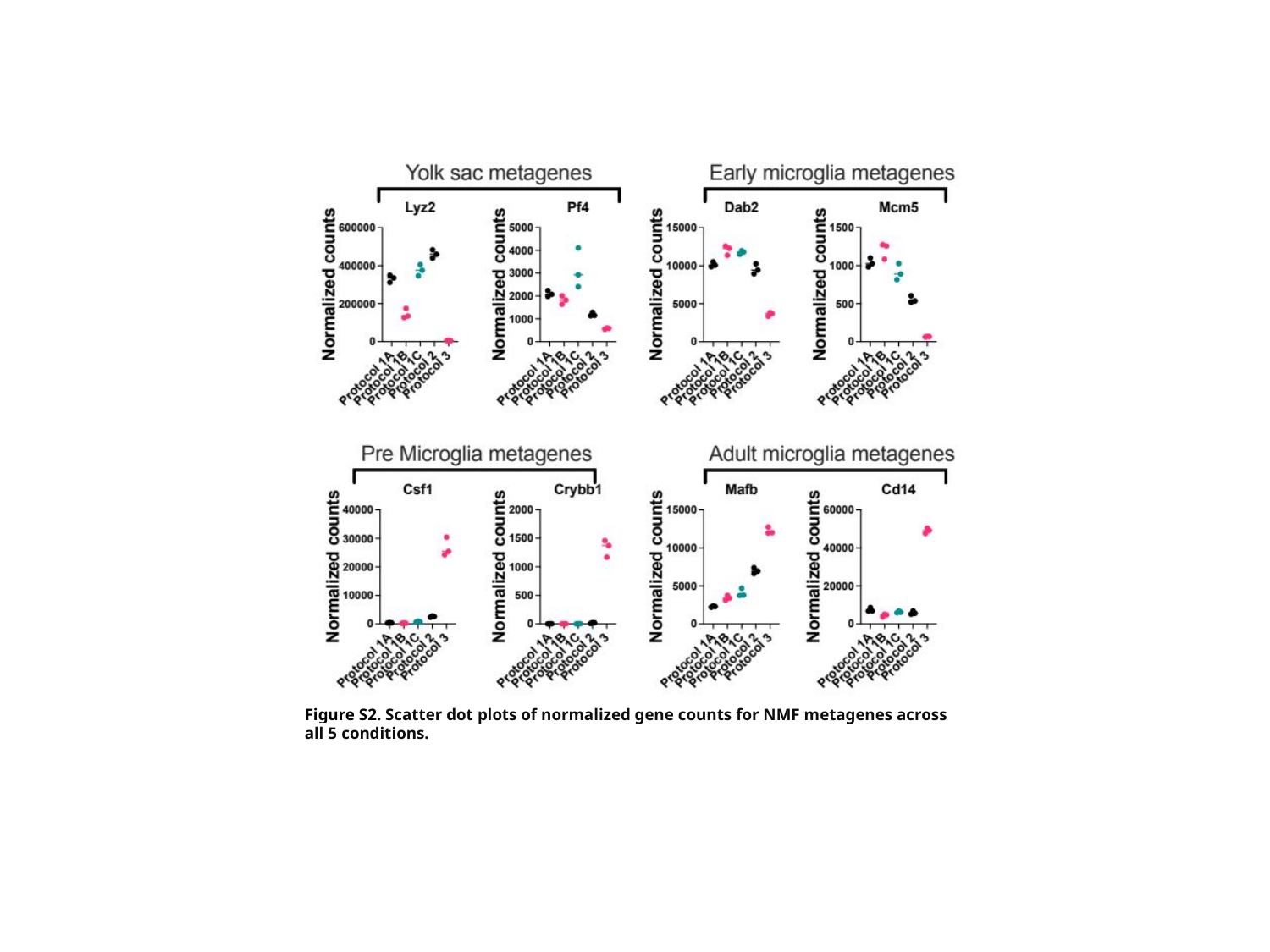

Figure S2. Scatter dot plots of normalized gene counts for NMF metagenes across all 5 conditions.
